# Impact of maternal glycemic control during pregnancy on early biomarkers of neonatal metabolic syndrome

**DOI:** 10.3389/fped.2026.1874461

**Published:** 2026-06-25

**Authors:** Xiaogang An, Yajun Kong

**Affiliations:** 1 Pediatric Department, Shanxi Province Fenyang Hospital, Fenyang, China; 2 Obstetrics Department, Shanxi Province Fenyang Hospital, Fenyang, China

**Keywords:** early biomarkers, gestational diabetes mellitus, maternal glycemic control, metabolic syndrome, neonate

## Abstract

**Objective:**

To investigate the effects of maternal glycemic control during pregnancy on early biomarkers of neonatal metabolic syndrome.

**Methods:**

We prospectively enrolled 130 pregnant women. Based on glycemic control, they were classified into adequate control group (*n* = 82, including 59 with normal glucose tolerance and 23 with well-controlled GDM) and poor control group (*n* = 48, GDM with suboptimal control). Neonatal tyrosine, alanine, total carnitine, C18 acylcarnitine, bilirubin, and hypoglycemia incidence were measured. Multivariable linear regression adjusted for confounders, with Bonferroni correction (*α*=0.01).

**Results:**

Compared with the adequate glycemic control group, neonates in the poor glycemic control group exhibited significantly lower levels of tyrosine and alanine (all *P* < 0.05), and significantly higher levels of total carnitine, C18 acylcarnitine, and bilirubin (all *P* < 0.05). The incidence of neonatal hypoglycemia was also significantly higher (*P* < 0.05). There were no significant differences between groups in the exact timing of blood sampling for amino acids or bilirubin (*P* > 0.05). After adjustment for pre-pregnancy body mass index (BMI), gestational age at delivery, mode of delivery, neonatal sex, maternal age, parity, feeding before blood collection, and thyroid dysfunction, poor maternal glycemic control remained independently associated with decreased neonatal tyrosine (*β* = −−0.335, *P* = 0.009) and alanine (*β* = −0.289, *P* = 0.019), as well as increased total carnitine (*β* = 0.381, *P* = 0.003), C18 acylcarnitine (*β* = 0.348, *P* = 0.006), and bilirubin levels (*β* = 0.405, *P* = 0.002). After Bonferroni correction for multiple comparisons (*α* = 0.01), tyrosine, total carnitine, C18 acylcarnitine, and bilirubin remained statistically significant, while alanine did not.

**Conclusions:**

Poor glycemic control during pregnancy is associated with multiple abnormalities in early neonatal metabolic biomarkers during the first 72 h after birth. Optimizing glycemic control during pregnancy may help mitigate early metabolic disturbances in the neonatal period. Future long-term follow-up studies are needed to determine whether these early biomarkers predict subsequent metabolic syndrome in later childhood or adulthood.

## Introduction

Abnormal glucose metabolism during pregnancy represents one of the most common metabolic complications in the perinatal period, with a steadily increasing global prevalence reaching 15%–20% in certain regions (1). Extensive population-based studies have shown that maternal hyperglycemia is associated with a higher incidence of both immediate adverse outcomes in offspring, for example, macrosomia, neonatal hypoglycemia, and respiratory distress syndrome, and long-term metabolic abnormalities such as obesity, insulin resistance, type 2 diabetes, and cardiovascular disease (2–4).

Metabolic syndrome involves a cluster of interrelated abnormalities, such as abdominal obesity, impaired glucose and lipid homeostasis, as well as elevated blood pressure. The underlying mechanisms of this condition are intimately associated with the concept of fetal programming. Existing research indicates that adverse intrauterine environments (especially maternal hyperglycemia) can induce lasting alterations in the structure and function of fetal organs through mechanisms such as epigenetic modification, mitochondrial dysfunction, and endocrine programming, thereby increasing susceptibility to metabolic diseases in later stages of life (5).

Recent advances in neonatal metabolomics have provided novel approaches for early identification of metabolic risk. Amino acids (e.g., tyrosine and branched-chain amino acids) and acylcarnitines are key metabolites reflecting energy metabolism and mitochondrial function. Existing research has shown that disruptions in tyrosine metabolic pathways are linked to insulin resistance. Furthermore, lower concentrations of tyrosine in the bloodstream are associated with a higher level of metabolic risk among pediatric populations (6). As intermediates in the process of fatty acid *β*-oxidation, acylcarnitines serve as sensitive biomarkers for mitochondrial dysfunction. Additionally, these metabolites contribute importantly to the early pathogenesis of both insulin resistance and nonalcoholic fatty liver disease (7).

Furthermore, neonatal bilirubin is involved in the regulation of oxidative stress and the maintenance of metabolic balance. While modest increases in bilirubin levels provide antioxidant benefits, deviations from the normal range may signal compromised metabolic adaptation (8). Therefore, combined assessment of amino acids, acylcarnitines, and bilirubin may facilitate the development of a sensitive biomarker panel for early prediction of metabolic syndrome.

Although an increasing body of evidence suggests that maternal hyperglycemia is associated with lasting metabolic consequences in offspring, there remains a paucity of clinical data concerning how specific levels of glycemic management influence early neonatal metabolic indicators. In particular, the distinct effects of satisfactory vs. inadequate blood glucose control on the metabolic profiles of newborns have yet to be clearly characterized.

Accordingly, the present prospective cohort study was designed to compare the concentrations of early indicators associated with metabolic syndrome—specifically, amino acids (tyrosine and alanine), acylcarnitines (free carnitine and octadecanoylcarnitine), as well as bilirubin—among neonates whose mothers achieved adequate glycemic control during pregnancy vs. those with poor control. Furthermore, the study sought to evaluate how maternal glycemic management levels influence these neonatal biomarkers. The results of this investigation are anticipated to offer evidence supporting the optimization of glycemic treatment strategies for early detection of metabolic alterations in offspring during the immediate postnatal period.

## Materials and methods

### Study design and ethics

The present investigation employed a single-center prospective cohort design aimed at assessing how maternal blood glucose management during gestation affects early metabolic syndrome biomarkers in newborns. All procedures adhered to the principles outlined in the Declaration of Helsinki (9), and approval for the study protocol was granted by the institutional ethics review board of Shanxi Province Fenyang Hospital. Prior to study entry, every participant provided written informed consent.

### Study population

#### Sample size calculation

Sample size determination was performed based on the primary outcome measure, namely the neonatal level of total carnitine. Drawing upon preliminary data and earlier investigations (10), we assumed an effect size of 0.8, along with a statistical power of 90% and a two-sided significance threshold of *α* = 0.05. Calculations conducted using PASS software indicated that a minimum of 40 participants would be necessary for each study group. To account for an anticipated attrition rate of roughly 10%, the target enrollment was raised to 45 individuals per group, resulting in an overall planned sample size of 90 participants. During the enrollment process, we observed that the proportion of women with poor glycemic control was lower than initially anticipated. To ensure that the poor glycemic control group reached the required minimum of 45 participants, we continued enrollment until this subgroup achieved an adequate sample size. Ultimately, when the poor glycemic control group reached 48 participants, the adequate glycemic control group had accumulated 82 participants, resulting in a final total of 130 mother-neonate pairs enrolled.

#### Inclusion and exclusion criteria

**Figure 1:**
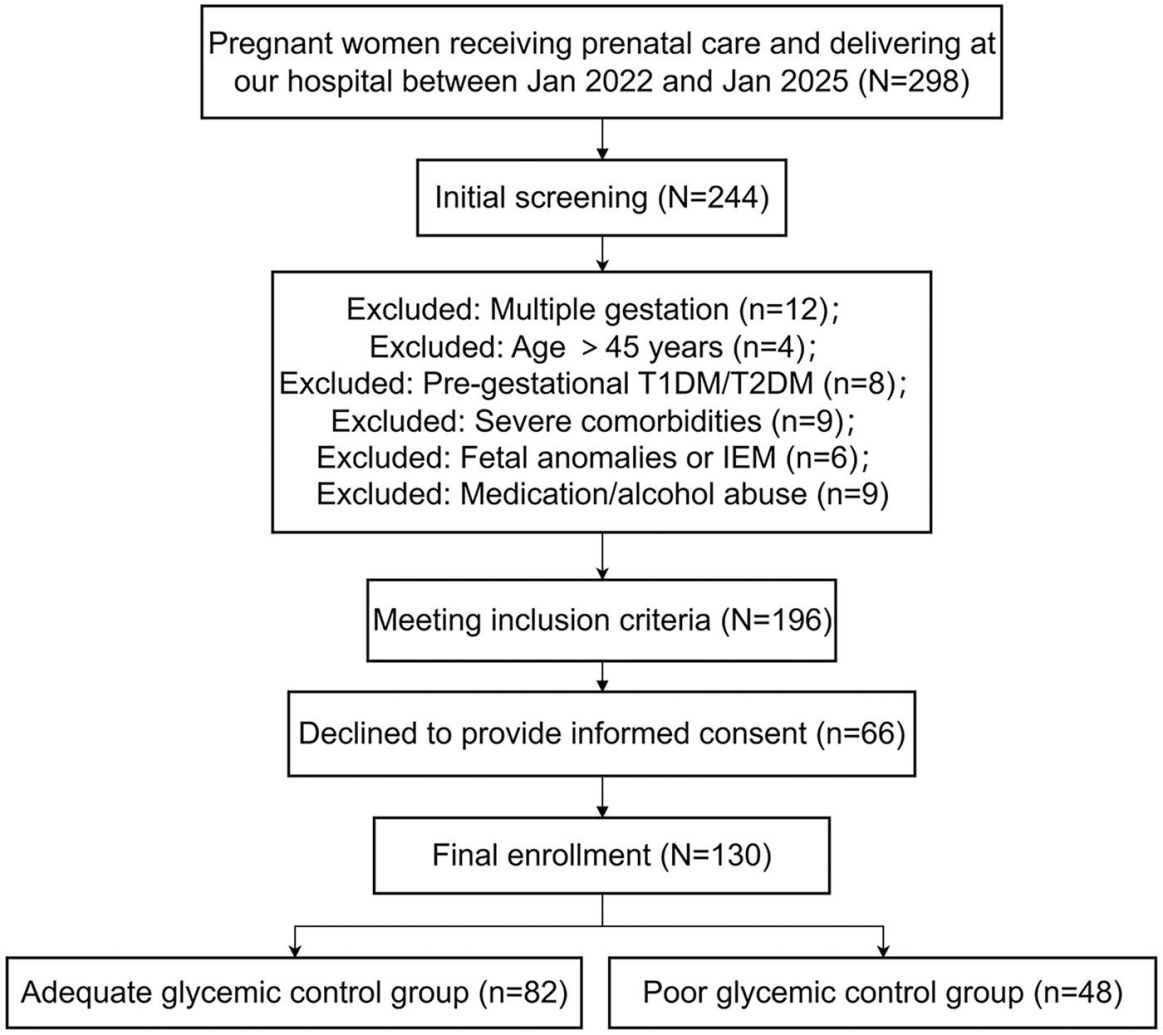
Flowchart of participant selection.

We consecutively enrolled pregnant women who underwent routine antenatal care and gave birth at our institution between January 2022 and January 2025. All enrolled individuals had established prenatal records at 11–13 weeks of gestation and were followed up until 72 h after delivery. The eligibility criteria were as follows: (1) pregnancy with a single fetus; (2) maternal age ranging from 18 to 45 years; (3) completion of both antenatal care and delivery at our hospital with comprehensive clinical documentation; (4) willingness to participate voluntarily and provision of signed informed consent. Exclusion criteria comprised: (1) a pre-pregnancy diagnosis of either type 1 or type 2 diabetes mellitus; (2) presence of severe hepatic or renal impairment, autoimmune conditions, malignant tumors, or thyroid dysfunction; (3) multiple-gestation pregnancy; (4) known fetal inborn errors of metabolism or major structural abnormalities; (5) administration of drugs known to substantially influence glucose metabolism during gestation (e.g., systemic glucocorticoids, excluding short-term local use); (6) a history of alcohol abuse or substance misuse. Following the screening process, 130 participants were enrolled, with 82 allocated to the adequate glycemic control arm and 48 to the poor glycemic control arm. A flowchart depicting the participant selection process is presented in Figure 1.

#### Classification of glycemic control

Between 24 and 28 weeks of gestation, each enrolled subject received a 75-g oral glucose tolerance test (OGTT). The diagnosis of gestational diabetes mellitus (GDM) followed the criteria established by the International Association of Diabetes and Pregnancy Study Groups (IADPSG). According to these standards, GDM is confirmed if any of the following plasma glucose thresholds are met or exceeded: a fasting value of 5.1 mmol/L, a 1-hour post-load value of 10.0 mmol/L, or a 2-hour post-load value of 8.5 mmol/L. Meeting at least one of these three criteria is sufficient for a GDM diagnosis (11). Based on their glycemic status during gestation, the enrolled women were divided into two categories. The first was the Adequate Glycemic Control Group, which consisted of individuals with normal glucose tolerance as well as those diagnosed with GDM who successfully met glycemic targets through standardized management protocols (encompassing medical nutrition therapy, physical exercise, and insulin treatment when necessary). Adequate control was operationally defined by the following criteria measured at 32–36 weeks of gestation: a fasting plasma glucose level below 5.3 mmol/L, a 2-hour postprandial glucose value below 6.7 mmol/L, and an HbA1c below 6.0%. The second category was the Poor Glycemic Control Group, comprising GDM-affected women who either did not receive standardized care or, despite receiving such care, failed to achieve the aforementioned glycemic goals. Poor control was defined by the presence of any of the following at 32–36 weeks: fasting plasma glucose at or above 5.3 mmol/L, 2-hour postprandial glucose at or above 6.7 mmol/L, or HbA1c at or above 6.0%.

All classifications were based on at least two follow-up measurements obtained between 24 weeks of gestation and delivery (interval ≥ 4 weeks).

#### Maternal clinical data collection

Structured questionnaires served as the tool for gathering clinical data, which encompassed demographic information (maternal age and gestational week at delivery), obstetric history including gravidity and parity, pre-pregnancy body mass index (BMI), behaviors during pregnancy such as active smoking or alcohol consumption (both considered as any reported use), and the presence of pregnancy-associated complications (for instance, hypertensive disorders of pregnancy or thyroid dysfunction).

Glycemic parameters, specifically FPG and 2hPPG, were assessed at multiple time points: early gestation (11–13 weeks and 6 days), mid-gestation (24–28 weeks), and late gestation (32–36 weeks, as well as within one week prior to delivery). These measurements were conducted using the hexokinase method on an automated biochemical analyzer (Hitachi 7,600, Japan). For individuals with a confirmed diagnosis of GDM, follow-up glucose assessments were repeated at intervals of two to four weeks until childbirth.

Within the week preceding delivery, samples of peripheral venous blood were obtained to determine HbA1c concentrations. This analysis was performed using high-performance liquid chromatography (Variant II Turbo system, Bio-Rad, USA), with results standardized according to NGSP protocols. HbA1c levels served as an indicator reflecting the average glycemic status over the preceding two to three months.

All data collection and laboratory procedures were conducted by uniformly trained research nurses. Data entry was performed independently by two researchers with logical verification.

### Measurement of neonatal metabolic biomarkers

#### Sample collection

During the first 24 h postpartum, peripheral blood obtained via heel lance or umbilical cord sampling was applied to specialized filter paper cards. Following room-temperature air-drying, these specimens were sealed and stored at a temperature of −80 °C for subsequent analysis of amino acids and acylcarnitines.

Separately, between 24 and 72 h after delivery, a volume of 1 mL of peripheral venous blood was drawn into vacuum tubes containing a clot activator. After being left undisturbed at ambient temperature for half an hour, the samples underwent centrifugation at 3,000 revolutions per minute for a duration of 10 min. The resulting serum was then isolated for bilirubin measurement.

#### Amino acid and acylcarnitine analysis

Quantitative analysis of amino acids (tyrosine and alanine) and acylcarnitines (total carnitine and stearoylcarnitine) in neonatal dried blood spots was performed using tandem mass spectrometry.

In brief, a disc measuring 3 mm in diameter was punched from each dried blood spot sample and then extracted using a solution spiked with isotope-labeled internal standards. This mixture was agitated at ambient temperature for a period of 20 min. Subsequently, the supernatant was transferred into a 96-well plate, evaporated under a stream of nitrogen, subjected to derivatization, reconstituted, and finally prepared for analytical measurement.

The analytical platform employed was an API 3,200 MD™ tandem mass spectrometer (manufactured by AB Sciex, USA), in conjunction with the NeoBase™ non-derivatized kit (produced by PerkinElmer, USA). Each step of the analytical process was carried out in strict compliance with the protocols specified by the manufacturer. Quality assurance was maintained by referencing the control ranges supplied within the kit documentation.

#### Bilirubin measurement

An automated biochemical analyzer (Hitachi 7,600, Japan) was employed to determine the concentrations of both total and direct bilirubin in serum samples. Assay reagents were obtained from Beijing Strong Biotechnologies, Inc. Daily internal quality control procedures were carried out, ensuring that the coefficient of variation remained under 5%.

#### Diagnostic criteria for neonatal hypoglycemia

The criterion for neonatal hypoglycemia was established as a blood glucose value falling below 2.6 mmol/L at any point during the first 24 h following delivery (12). Blood glucose levels were confirmed using both a bedside glucometer (Johnson & Johnson OneTouch®) and a biochemical analyzer.

#### Outcome measures

The primary outcome was the difference in early biomarkers of neonatal metabolic syndrome (tyrosine, alanine, total carnitine, and stearoylcarnitine) between the two groups.

Secondary outcomes included serum total bilirubin levels within 24–72 h after birth, the incidence of neonatal hypoglycemia, and the independent effect of maternal glycemic control during pregnancy on neonatal metabolic biomarkers.

### Statistical analysis

All statistical evaluations were carried out using SPSS, version 26.0. For continuous variables exhibiting a normal distribution, data were reported as mean values along with their standard deviations (mean ± SD), and group comparisons were performed via the independent samples t-test. In cases where continuous variables did not follow a normal distribution, results were summarized as medians accompanied by interquartile ranges [M (Q1, Q3)], with group differences assessed using the Mann–Whitney U test. Categorical variables were expressed as frequencies accompanied by percentages, and comparisons between groups were made using either the chi-square test or Fisher's exact test, as appropriate. To examine the independent influence of maternal glycemic control on neonatal metabolic biomarkers, a multiple linear regression model was employed, adjusting for pre-pregnancy BMI, gestational age at delivery, mode of delivery (vaginal vs. cesarean), neonatal sex, maternal age, parity, feeding before blood collection (yes/no), and maternal thyroid dysfunction (yes/no) as covariates. Given that five primary metabolic outcomes were examined (tyrosine, alanine, total carnitine, C18 acylcarnitine, bilirubin), we applied the Bonferroni correction for multiple comparisons, setting the statistical significance threshold at *α* = 0.05/5 = 0.01. Both unadjusted and Bonferroni-corrected *P* values are reported. A two-sided *P* value below 0.05 was regarded as indicating statistical significance for secondary outcomes and baseline comparisons; for primary outcomes, the Bonferroni-corrected threshold of 0.01 was applied.

## Results

### Comparison of baseline characteristics

A total of 130 mother–neonate pairs were ultimately enrolled in the present study, with 82 assigned to the adequate glycemic control arm (59 with normal glucose tolerance and 23 with GDM who achieved glycemic targets) and 48 to the poor glycemic control arm. As summarized in Table 1, no statistically significant between-group differences were observed regarding maternal age, parity, history of smoking, history of alcohol consumption, or pregnancy-related complications such as hypertensive disorders of pregnancy and thyroid dysfunction (all *P* > 0.05). There were also no significant differences in the distribution of mode of delivery, neonatal sex, or feeding before blood collection between the two groups (all *P* > 0.05). However, when compared with the adequate glycemic control group, women in the poor glycemic control group demonstrated a significantly higher pre-pregnancy body mass index (*P* < 0.05) as well as a notably earlier gestational week at the time of delivery (*P* < 0.05).

**Table 1:** Comparison of baseline characteristics between the two groups (mean ± SD).

Variable	Adequate glycemic control group (*n* = 82)	Poor glycemic control group (*n* = 48)	*t/χ^2^* value	*P* value
Age (years)	30.5 ± 4.2	31.2 ± 4.5	−0.892	0.374
Pre-pregnancy BMI (kg/m^2^)	22.3 ± 2.8	25.1 ± 3.2	−5.214	＜0.001
Gestational age at delivery (weeks)	39.1 ± 1.2	38.2 ± 1.5	3.756	＜0.001
Parity (primipara/multipara, n, %)	45（54.9）/37（45.1）	26（54.2）/22（45.8）	0.006	0.937
Mode of delivery (vaginal/cesarean, n, %)	51（62.2）/31（37.8）	28（58.3）/20（41.7）	0.642	0.423
Neonatal sex (male/female, n, %)	43（52.4）/39（47.6）	25（52.1）/23（47.9）	0.168	0.682
Feeding before blood collection (yes/no, n, %)	18（22.0）/64（78.0）	12（25.0）/36（75.0）	0.874	0.350
Smoking history (yes/no, n, %)	3（3.7）/79（96.3）	2（4.2）/46（95.8）	0.107	0.744
Alcohol consumption history (yes/no, n, %)	2（2.4）/80（97.6）	1（2.1）/47（97.9）	0.225	0.635
Hypertensive disorders of pregnancy (n, %)	5（6.1）	6（12.5）	0.882	0.348
Thyroid dysfunction (n, %)	4（4.9）	5（10.4）	0.710	0.399

### Comparison of blood sampling timing

The mean age at blood collection for amino acid analysis was 14.2 ± 5.1 h in the adequate control group vs. 14.8 ± 5.4 h in the poor control group (*P* = 0.528). For bilirubin measurement at 24–72 h, the mean sampling time was 48.3 ± 12.5 h vs. 49.1 ± 13.2 h, respectively (*P* = 0.731). These differences were not statistically significant.

### Comparison of blood glucose and HbA1c levels during pregnancy

Table 2 presents the comparison between the two groups. Relative to those with adequate glycemic control, pregnant women classified under poor glycemic control exhibited markedly elevated fasting plasma glucose levels during the first, second, and third trimesters. Additionally, this group showed significantly higher two-hour postprandial glucose values in both the second and third trimesters, along with increased HbA1c concentrations prior to delivery (all comparisons yielded *P* < 0.05).

**Table 2:** Comparison of blood glucose and HbA1c levels during pregnancy (mean ± SD).

Indicator	Adequate glycemic control group (*n* = 82)	Poor glycemic control group (*n* = 48)	*t* value	*P* value
Early pregnancy FPG (mmol/L)	4.8 ± 0.4	5.3 ± 0.6	−5.628	＜0.001
Mid-pregnancy FPG (mmol/L)	4.9 ± 0.5	5.6 ± 0.7	−6.743	＜0.001
Late pregnancy FPG (mmol/L)	5.0 ± 0.4	5.8 ± 0.8	−7.455	＜0.001
Mid-pregnancy 2hPPG (mmol/L)	6.8 ± 1.0	8.9 ± 1.4	−9.862	＜0.001
Late pregnancy 2hPPG (mmol/L)	6.5 ± 0.9	8.5 ± 1.3	−10.124	＜0.001
HbA1c before delivery (%)	5.4 ± 0.3	6.4 ± 0.5	−14.056	＜0.001

### Comparison of early markers of neonatal metabolic syndrome and incidence of hypoglycemia

Table 3 reveals that, relative to the adequate glycemic control group, neonates whose mothers experienced poor glycemic control exhibited markedly reduced concentrations of both tyrosine and alanine (*P* < 0.05). Conversely, this group showed significantly elevated levels of total carnitine, stearoylcarnitine (an 18-carbon acylcarnitine), and total bilirubin measured between 24 and 72 h postpartum, along with a greater frequency of neonatal hypoglycemia (*P* < 0.05).

**Table 3:** Comparison of early markers of neonatal metabolic syndrome and incidence of hypoglycemia.

Indicator	Adequate glycemic control group (*n* = 82)	Poor glycemic control group (*n* = 48)	*t/χ^2^* value	*P* value
Amino acids (*μ*mol/L)				
Tyrosine	98.5 ± 15.2	82.3 ± 12.8	6.134	＜0.001
Alanine	245.6 ± 32.4	218.9 ± 28.7	4.672	＜0.001
Acylcarnitines (μmol/L)				
Total carnitine	28.4 ± 5.6	35.2 ± 6.8	6.178	＜0.001
Stearoylcarnitine (C18)	0.18 ± 0.04	0.23 ± 0.05	6.298	＜0.001
Total bilirubin at 24–72 h after birth (μmol/L)	145.3 ± 28.6	168.7 ± 32.4	4.324	＜0.001
Neonatal hypoglycemia (n, %)	4（4.9）	10（20.8）	6.446	0.011

### Multivariate linear regression analysis of the effects of glycemic control during pregnancy on neonatal metabolic markers

Using glycemic control during pregnancy (poor glycemic control = 1, good glycemic control = 0) as the independent variable and each neonatal metabolic marker as the dependent variable, multivariate linear regression analysis was performed after adjusting for pre-pregnancy BMI, gestational age at delivery, mode of delivery, neonatal sex, maternal age, parity, feeding before blood collection, and thyroid dysfunction. As shown in Table 4, poor glycemic control during pregnancy was independently associated with decreased levels of neonatal tyrosine and alanine, and independently associated with increased levels of total carnitine, stearoylcarnitine, and bilirubinafter Bonferroni correction for multiple comparisons (*α* = 0.01). The association with decreased alanine levels (*P* = 0.019) did not reach statistical significance after correction.

**Table 4:** Multivariate linear regression analysis of the effects of glycemic control during pregnancy on neonatal metabolic markers.

Dependent variable	*β*(95% CI)	Standard error	Standardized β	*P* value
Tyrosine (μmol/L)	−15.52 (−27.18, −3.86)	5.86	−0.335	0.009
Alanine (μmol/L)	−27.58 (−50.34, −4.82)	11.42	−0.289	0.019
Total carnitine (μmol/L)	6.71 (2.48, 10.94)	2.14	0.381	0.003
Stearoylcarnitine (μmol/L)	0.05 (0.02, 0.08)	0.02	0.348	0.006
Bilirubin (μmol/L)	22.98 (9.15, 34.81)	6.48	0.405	

Adjusted for pre-pregnancy BMI, gestational age at delivery, mode of delivery, neonatal sex, maternal age, parity, feeding before blood collection, and maternal thyroid dysfunction. After Bonferroni correction for five primary outcomes (*α* = 0.01), tyrosine, total carnitine, stearoylcarnitine, and bilirubin remained statistically significant; alanine did not.

## Discussion

This prospective cohort study, conducted at a single center, examined the relationship between the level of glycemic management achieved by mothers during gestation and the presence of metabolic-syndrome-related biomarkers in their newborns during the early postnatal period. The results showed that, compared with neonates born to mothers with adequate glycemic control, those born to mothers with poor glycemic control exhibited significantly lower tyrosine and alanine levels, significantly higher total carnitine, C18 acylcarnitine, and total bilirubin levels within 24–72 h after birth, as well as a significantly higher incidence of neonatal hypoglycemia. Furthermore, after adjustment for prepregnancy BMI and gestational age at delivery, poor maternal glycemic control remained independently associated with these metabolic abnormalities. These findings suggest that intrauterine exposure to hyperglycemia may induce early metabolic programming alterations in the offspring by disturbing amino acid metabolism, fatty acid beta-oxidation, and bilirubin metabolism, thereby contributing to a neonatal metabolic phenotype that may be associated with future metabolic syndrome risk, although direct evidence from this 72-hour study is not available.

Our findings revealed that newborns belonging to the poor glycemic control group exhibited notably reduced concentrations of both tyrosine and alanine compared to their counterparts. Tyrosine is a precursor for catecholamine synthesis and plays an important role in stress response and energy metabolism regulation, whereas alanine is a key substrate for gluconeogenesis and is essential for maintaining glucose homeostasis in the neonatal period. Previous studies have suggested that maternal hyperglycemia can overstimulate fetal pancreatic beta cells, resulting in fetal hyperinsulinemia, which in turn promotes peripheral amino acid uptake and utilization and accelerates the consumption of gluconeogenic substrates (13–15). In addition, hyperinsulinemia suppresses hepatic endogenous glucose production, thereby increasing the compensatory demand for conversion of gluconeogenic precursors such as alanine into glucose, which may further reduce their circulating concentrations (15). Our findings are in line with these mechanisms and indicate that poor maternal glycemic control may lead to early depletion of neonatal amino acid reserves through hyperinsulinemia-mediated metabolic reprogramming. Such alterations may represent an early metabolic vulnerability linked to future glucose intolerance and type 2 diabetes in the offspring. The finding of decreased tyrosine and alanine differs from the elevated branched-chain amino acids typically seen in adult insulin resistance. In the context of fetal hyperinsulinemia induced by maternal hyperglycemia, insulin promotes peripheral amino acid uptake and suppresses hepatic gluconeogenesis, increasing the demand for gluconeogenic precursors such as alanine (15). Thus, the decreased levels likely reflect increased peripheral utilization rather than impaired synthesis, a mechanism supported by Zheng et al. (14).

Acylcarnitines are intermediate products of fatty acid beta-oxidation, and changes in their profiles can reflect mitochondrial fatty acid oxidation status. In this study, neonates in the poor glycemic control group had significantly higher total carnitine and C18 acylcarnitine levels. Carnitine in its total form plays an essential role in shuttling long-chain fatty acids across the mitochondrial membrane to undergo beta-oxidation. An increase in its concentration might reflect a compensatory upregulation of fatty acid oxidation pathways (16). C18 acylcarnitine, a representative long-chain acylcarnitine species, generally accumulates in the setting of incomplete mitochondrial beta-oxidation or impaired fatty acid oxidation (17). In the setting of maternal hyperglycemia, the fetus experiences heightened exposure to both glucose and free fatty acids. This increased availability of lipid substrates may impose an elevated metabolic load on tissues with high energy demands, including the heart, skeletal muscle, and liver. Should the capacity for fatty acid oxidation prove inadequate, long-chain acylcarnitines undergo incomplete metabolism, resulting in their subsequent accumulation within the bloodstream (18). Moreover, hyperinsulinemia may inhibit carnitine palmitoyltransferase-1 activity, further limiting mitochondrial entry of long-chain fatty acids and contributing to the elevation of long-chain acylcarnitines such as C18 acylcarnitine (19). Accordingly, the altered acylcarnitine profile observed in our study may indicate the presence of early fatty acid metabolic dysregulation in neonates exposed to poor maternal glycemic control, which may constitute part of the molecular basis for later insulin resistance, nonalcoholic fatty liver disease, and metabolic syndrome. C18 acylcarnitine (stearoylcarnitine) was selected as a marker of incomplete mitochondrial *β*-oxidation. In maternal hyperglycemia, the fetus experiences increased glucose and free fatty acid fluxes, leading to lipid substrate overload in mitochondria. When *β*-oxidation capacity is exceeded, long-chain acylcarnitines such as C18 accumulate, indicating early fetal metabolic malprogramming characterized by mitochondrial fatty acid oxidation inefficiency (17, 18).

We also found that neonates in the poor glycemic control group had significantly higher total bilirubin levels at 24–72 h after birth and a significantly increased incidence of neonatal hypoglycemia. Hyperbilirubinemia is common among neonates born to mothers with gestational diabetes mellitus, and several mechanisms may explain this association. First, chronic intrauterine hypoxia induced by maternal hyperglycemia may stimulate erythropoietin secretion and lead to polycythemia, thereby increasing bilirubin production after birth because of enhanced red blood cell breakdown (20). Second, fetal hyperinsulinemia may impair hepatic bilirubin uptake and reduce the activity of uridine diphosphate glucuronosyltransferase, thereby decreasing bilirubin conjugation and excretion (21). In addition, earlier gestational age at delivery, which was also observed in the poor glycemic control group in the present study, is itself an independent risk factor for neonatal hyperbilirubinemia. The increased risk of neonatal hypoglycemia is more directly attributable to the persistent effect of fetal hyperinsulinemia. After cord clamping, the maternal glucose supply ceases abruptly, whereas circulating insulin levels may remain elevated, leading to accelerated peripheral glucose utilization and suppression of glycogenolysis and gluconeogenesis, ultimately resulting in hypoglycemia (22). Because recurrent or severe hypoglycemia may cause irreversible neurodevelopmental injury, neonates born to mothers with poor glycemic control should undergo close postnatal glucose monitoring and timely feeding intervention.

Our results are generally consistent with previous reports. Zheng et al. (14) demonstrated altered amino acid metabolic profiles in neonates born to mothers with gestational diabetes mellitus, with lower alanine levels being negatively associated with the risk of neonatal hypoglycemia. With respect to acylcarnitines, Awad et al. (23) similarly reported elevated total carnitine and long-chain acylcarnitines in neonates exposed to gestational diabetes mellitus, suggesting adaptive changes in fetal fatty acid oxidation. Regarding bilirubin metabolism, Xu et al. (24) identified gestational diabetes mellitus as an independent risk factor for neonatal hyperbilirubinemia (OR = 5.356, *P* = 0.03) using multivariable logistic regression analysis. The main strength of the present study is that multiple metabolic markers were integrated into a unified analytical framework, and the independent effect of poor maternal glycemic control was confirmed using multivariable regression models. This provides more comprehensive evidence linking maternal glycemic status during pregnancy to early-life metabolic programming in the offspring. This study employed a targeted metabolomics approach based on *a priori* hypotheses from existing literature, rather than untargeted screening. The selected biomarkers—tyrosine, alanine, total carnitine, C18 acylcarnitine, and bilirubin—have each been previously implicated in fetal programming or neonatal metabolic risk (6, 8, 15–17). This focused approach reduces the risk of false discoveries from multiple comparisons.

Several limitations of the present work warrant consideration. First, because this investigation was conducted at a single institution, the demographic and regional features of the study population may restrict the applicability of our results to other settings. To confirm the external validity of these observations, multicenter studies incorporating larger cohorts are necessary. Second, while key confounding variables—namely pre-pregnancy BMI, gestational age at delivery, and thyroid dysfunction—were accounted for in our regression models, the potential influence of unmeasured factors, such as maternal dietary habits, physical activity levels, and genetic predisposition, cannot be ruled out. Third, the adequate glycemic control group included a majority of women with normal glucose tolerance (59/82, 71.9%) and only 23 with well-controlled GDM; therefore, the observed differences may partly reflect the presence or absence of GDM itself rather than solely the quality of glycemic control. Future studies should compare well-controlled GDM vs. poorly controlled GDM directly. Fourth, the relatively modest sample size in the poor glycemic control arm (*n* = 48) and the imbalance between groups may have constrained the statistical power available for subgroup analyses. Fifth, our investigation concentrated solely on metabolic disturbances during the immediate neonatal phase, without incorporating extended longitudinal follow-up. Consequently, a direct causal link between these early biomarkers and subsequent metabolic syndrome remains unestablished. Future prospective cohort studies with long-term follow-up are needed to assess whether neonatal metabolic markers can predict metabolic outcomes in childhood and beyond. Sixth, the absence of cord blood insulin and C-peptide measurements prevents direct confirmation of whether fetal hyperinsulinemia serves as a mediating factor. Seventh, although thyroid dysfunction was adjusted for in the multivariable model, we cannot completely exclude residual confounding from subclinical thyroid dysfunction or variation in thyroid hormone levels within the normal range. This proposed mechanistic pathway should be explored in subsequent research endeavors.

## Conclusions

In summary, poor maternal glycemic control during pregnancy is an independent risk factor for early metabolic abnormalities in neonates within the first 72 h after birth. These alterations are characterized by reduced amino acid levels, accumulation of acylcarnitines, elevated bilirubin levels, and increased risk of neonatal hypoglycemia, reflecting coordinated disturbances in energy metabolism in the immediate neonatal period.

These findings extend the understanding of maternal hyperglycemia beyond traditional perinatal complications and highlight its role in early-life metabolic programming. Effective glycemic management during pregnancy, including strict control of fasting glucose, postprandial glucose, and HbA1c within recommended targets, may not only improve short-term outcomes but also reduce early metabolic disturbances in the neonatal period. Whether these improvements translate into reduced long-term cardiometabolic risk in offspring requires further investigation with extended follow-up.

## Data Availability

The original contributions presented in the study are included in the article/Supplementary Material, further inquiries can be directed to the corresponding author.
